# Early childhood adversity and body mass index in childhood and adolescence: linking registry data on adversities with school health records of 53,401 children from Copenhagen

**DOI:** 10.1038/s41366-023-01355-9

**Published:** 2023-08-25

**Authors:** Leonie K. Elsenburg, Andreas Rieckmann, Jessica Bengtsson, Theis Lange, Jennifer L. Baker, Thorkild I. A. Sørensen, Naja Hulvej Rod

**Affiliations:** 1 Section of Epidemiology, Department of Public Health, University of Copenhagen, Copenhagen, Denmark; 2 Section of Biostatistics, Department of Public Health, University of Copenhagen, Copenhagen, Denmark; 3 Section of Epidemiology, Center for Clinical Research and Prevention, Copenhagen University Hospital - Bispebjerg and Frederiksberg, Copenhagen, Denmark; 4 Section on Genomic Physiology and Translation, Novo Nordisk Foundation Center for Basic Metabolic Research, University of Copenhagen, Copenhagen, Denmark

**Keywords:** Risk factors, Obesity, Epidemiology, Paediatrics

## Abstract

**Objective:**

We examined whether childhood adversity experienced in early childhood (0–5 years) is related to body mass index (BMI) in childhood (6–7 years) and adolescence (12–15 years).

**Methods:**

This study combined data from the nationwide register-based DANLIFE study on childhood adversities with data on height and weight of school children in Copenhagen. Data were available for 53,401 children born in Denmark between 1980 and 1996. Children were divided into groups of early childhood adversity by applying group-based multi-trajectory modelling using their yearly count of childhood adversity in three dimensions (i.e., material deprivation, loss or threat of loss, and family dynamics) from 0–5 years. Direct and total associations between the early childhood adversity groups and BMI z-scores in childhood and adolescence were estimated using sex-stratified structural equation models.

**Results:**

Five exclusive and exhaustive groups of early childhood adversity were identified, which were characterized by low adversity (51%), moderate material deprivation (30%), high material deprivation (14%), loss or threat of loss (3%) and high adversity (2%). Boys and girls exposed to moderate or high material deprivation and loss or threat of loss had a slightly higher BMI z-score, especially in adolescence, compared with those in the low adversity group, with the strongest association found for girls in the loss or threat of loss group (b (95% CI) = 0.18 (0.10, 0.26)). Additionally, boys in the high adversity group had a slightly lower BMI z-score in childhood than boys in the low adversity group (b (95% CI) = −0.12 (−0.22, −0.02)).

**Conclusions:**

Whereas associations with BMI were found for children and adolescents exposed to material deprivation, loss or threat of loss, and high adversity, the effect sizes were generally small. Contrary to prevailing hypotheses, weight changes in childhood is probably not a major explanatory mechanism linking early childhood adversity with later-life morbidity.

## Introduction

Childhood adversity is related to a sizeable burden of morbidity and mortality throughout the life course [1, 2]. It entails experiences such as economic hardship, physical or mental illness in the family, and parental divorce. Using large-scale life course data, we have recently shown that the experience of adversity is related to health and hospitalization patterns already in childhood [3], but we need further insights into the underlying mechanisms. Childhood adversity may affect health behaviours (e.g., physical activity, diet, and sleep behaviours) and lead to a physiological stress response with associated inflammatory processes and altered metabolism [4, 5]. Childhood adversity may lead to weight changes and differences in body mass index (BMI) during childhood. Childhood overweight and obesity, defined as a high BMI for age and sex, have also been shown to be related to a higher risk of morbidity and mortality in adulthood [6]. Childhood BMI may constitute an important factor linking adversity with later-life health as well as an early target point for intervention, which needs further exploration.

Systematic reviews have assessed the association between childhood adversity, in isolation or accumulated, and childhood BMI or obesity [7–9]. Findings were mixed with one review concluding there was no clear association between childhood maltreatment and obesity in children and adolescents [7] and two other reviews suggesting a positive association between adverse events and BMI, overweight and obesity in childhood [8, 9]. The mixed results of these previous studies may be due to differences in the definition and measurement of childhood adversity, the time frame in which childhood adversity and changes in BMI were studied, and the included study populations. To advance our understanding of the relationship between childhood adversity and BMI in childhood, there is a need for longitudinal studies including large, unselected samples in which sound methods are used to assess childhood adversity and BMI [9]. In addition, the type of adversity experienced, and the timing and the duration of exposure are important factors to study to deepen our understanding of the relationship [10, 11].

We add to this line of research by using a unique combination of data sources, consisting of register data on all children born in Denmark between 1980 and 1996 and health examination data of all children attending schools in the Copenhagen municipality (the capital of Denmark). Using this combination of data sources, we examine whether childhood adversity experienced before school age in Denmark (0–5 years) is related to body mass index at school entry (6–7 years) and in adolescence (12–15 years). BMI in the early school years is from after the adiposity rebound, which is the rise in BMI that occurs around age 6 [12], whereas BMI in adolescence is more predictive of adult BMI and weight status than childhood BMI and weight status [13, 14]. Combined, these data sources allow us to draw inferences based on 53,401 children for which objective information on exposure to a wide range of childhood adversities as well as on height and weight is available. As both childhood adversity and height and weight are measured objectively, the results of this study cannot be influenced by recall bias. The data additionally allow us to assess the influence of the type and the level of adversity experienced, as well as the influence of the studied time frame for the identification of differences in BMI and BMI changes in an unselected sample.

## Methods

For this study, we used data from the Danish life course (DANLIFE) cohort and the Copenhagen School Health Records Register (CSHRR). DANLIFE includes information on childhood adversities experienced by all children born in Denmark between 1 January 1980 and 31 December 2015 [15]. The CSHRR contains information from universal health examinations of school children in the central municipality of Copenhagen who are born between 1930 and 1996 [16]. At these health examinations, children’s height and weight were measured by a trained school health doctor or nurse. The sample for this study consists of all children (1) who are in both DANLIFE and the CSHRR, (2) who did not die or emigrate before their 6th birthday and (3) who have information on height and weight at 6-7 years and/or 12–15 years (*n* = 53,401). The processing of personal data for statistical or scientific purposes in the DANLIFE study is approved on behalf of the Danish Data Protection Agency by the Faculty of Health and Medical Sciences at the University of Copenhagen (Copenhagen, Denmark) (record number 514-0641/21-3000). The Danish Data Protection Agency ensures compliance with national and EU legislation. No ethical approval was obtained for this study, as it is not required for register-based studies according to Danish law.

### Early childhood adversity

To derive early childhood adversity groups, we applied group-based multi-trajectory modelling (using the package *traj* for Stata) to the yearly count of adversities children experienced from 0–5 years across three dimensions [17]. The three dimensions were material deprivation (i.e., family poverty and parental long-term unemployment), loss or threat of loss (i.e., parental and sibling somatic illness and death), and family dynamics (i.e., foster care placements, maternal separation, sibling psychiatric illness, and parental psychiatric illness, alcohol abuse and drug abuse). Definitions of the included childhood adversities can be found in Supplementary Table 1. We employed the same method as in an earlier study based on DANLIFE data [2], where we used zero-inflated Poisson regressions with a cubic trajectory function to model childhood adversity from 0–15 years. However, in this study we fitted the trajectories using an age span of 0–5 years. Based on the findings of the previous study, in which we identified 5 groups, we tested between 4 and 6 adversity groups to identify the optimal number of groups in this study. The optimal number of groups was based on interpretability and the percentage of individuals in each group, i.e., solutions including very small groups were disregarded [17, 18]. All children were allocated to the group they were most likely to belong to. In a post-hoc analysis, we performed the main analysis weighted by children’s posterior probabilities.

### Body mass index

Children were examined by school health doctors or nurses throughout their school career as prescribed by law. The first preventative health examination was in the first year of school (between age 5 and 7 years) and the last examination took place before the child graduated compulsory secondary education (before age 16 years). To determine childhood BMI, we used height and weight measurements taken at children’s ages 6–7 years. We prioritized the measurements closest to age 7 for children with multiple measurements, because children at this age are more likely to have experienced the adiposity rebound than at an earlier age [12]. For adolescent BMI, we selected height and weight measurements at ages 12–15 years and we prioritized the measurements closest to age 14 years, as most children will have height and weight measurements around this age.

Using children’s height and weight, we calculated their BMI. BMI was calculated by dividing weight (kg) by height (m) squared. As children’s BMI changes naturally with age, and differently for boys and girls, we used sex-specific BMI reference curves over age to convert children’s BMI to BMI z-scores, also called BMI standard deviation scores. We created these BMI reference curves by using the CSHRR data from all children born 1 January 1980 until 31 December 1996 (*n*_boys_ = 32,875; *n*_girls_ = 31,540). These reference curves were created by modelling BMI over age using the LMS method. The LMS method bases the reference curves on the skewness (L), median (M) and coefficient of variation (S) of the BMI data over age [19]. The reference curves were created separately for boys and girls, using the lms.bcn function in the R package VGAM. Based on these reference curves and the child’s BMI, sex and age, we determined the child and adolescent BMI z-scores.

### Covariates

Potential confounders of the association between childhood adversity and BMI included maternal age at birth [20], parental region of origin [4], parental cardiometabolic illness [21] and year of birth. All information regarding the covariates was extracted from Danish nationwide registers. Maternal age at birth was classified as younger (<20 years), average (20–30 years), or older (>30 years). Parental origin was classified as Western when one or both parents had a European, North American, Australian or New Zealand nationality, and as non-Western if both parents had another nationality. Parental cardiometabolic illness (yes/no) in the three years before birth of the child was established by collecting information on both primary and secondary diagnoses and primary cause-specific mortality. Parental cardiometabolic illness in the three years before birth of the child was considered present if parents were registered in the Danish National Patient Register or the Danish Register of Causes of Death with ischaemic heart disease (ICD8: 410–414/ICD10: I20.0, I20.1, I21–I25), cerebrovascular disease (430–438/I60–I69), congestive heart failure (427.09–427.11, 427.19, 428.99, 782.49/I50, I11.0, I13.0, I13.2), peripheral vascular disease (440–445/I70–I74, I77), type 1 diabetes (249/E10), or type 2 diabetes (250/E11) [22, 23].

In additional analyses, we adjusted for parental education at birth, size for gestational age, and preterm birth. Parental education at birth was classified as low (<10 years), medium (10–12 years), and high (>12 years). Size for gestational age at birth was based on percentiles of age- and sex-specific intrauterine growth reference curves and divided into small (<10th), average (≥10th to ≤90th), and large (>90th) [24]. Preterm birth (yes/no) was classified as childbirth <37 weeks of gestation.

### Statistical analysis

**Fig. 1:**
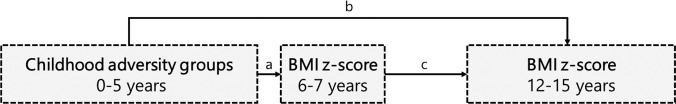
Graphical presentation of the examined associations between childhood adversity groups and BMI z-score in childhood and adolescence. The (direct) association of childhood adversity groups with BMI z-score in childhood (a), the direct association of childhood adversity groups with BMI z-score in adolescence (b), and the total association of childhood adversity groups with BMI z-score in adolescence (a*c + b).

We performed structural equation modelling to assess the associations between the childhood adversity groups and BMI z-score in (1) childhood and (2) adolescence (Fig. 1), using the group with the lowest exposure to childhood adversity as the reference. We examined the direct associations in childhood (a) and adolescence (b) and the total association in adolescence (a*c + b). The analyses were stratified by sex, as there may be sex differences in the association between childhood adversity and BMI [9]. In the main analysis, we adjusted for parental origin, maternal age at birth, parental cardiometabolic illness in the three years before birth, and year of birth. In an additional analysis, we further adjusted for parental educational level at birth. We adjusted for parental education at birth separately, as it is closely related to the material deprivation dimension of our childhood adversity groups and adjusting for parental educational level could therefore be considered as overadjusting for dimensions of social and family-related adversity. In a second additional analysis, we further adjusted for size for gestational age and preterm birth as they may have been influenced by social and family-related adversity before birth and could therefore be considered mediators of the association between early life adversity and health instead of confounders. In a sensitivity analysis, we performed the main analysis unadjusted for parental cardiometabolic illness in the three years before birth. Structural equation modelling was performed in STATA (version 14) using the sem command and mlmv (maximum likelihood with missing values), which uses full information maximum likelihood (FIML) to handle missing data.

## Results

### Early childhood adversity groups

**Fig. 2:**
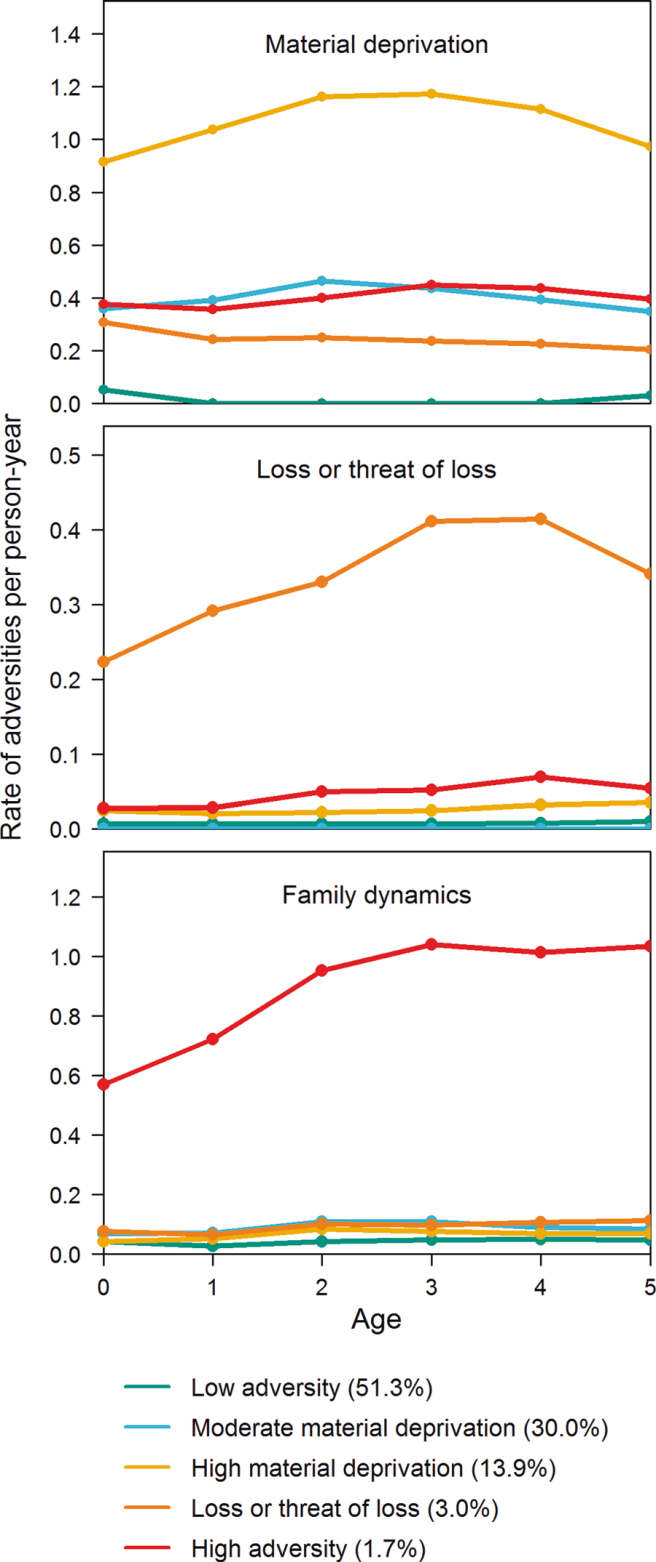
Childhood adversity groups from age 0–5 years. Rates of adversities in each of the five childhood adversity groups are shown across the three dimensions (material deprivation, loss or threat of loss, and family dynamics).

We considered five groups of childhood adversity from 0–5 years to be the optimal number of groups (Fig. 2 and Supplementary Table 2). About half of the children (51%) was in the *low adversity* group characterized by low rates of adversity across all three dimensions from 0–5 years. Thirty percent was in the *moderate material deprivation* group characterized by moderately elevated rates of material deprivation throughout early childhood, but low rates of adversity in the other dimensions. Fourteen percent of children was in the *high material deprivation* group characterized by particularly high rates of material deprivation in early childhood, but low rates of adversity in the other dimensions. A small proportion of the children (3%) was in the *loss or threat of loss* group characterized by high rates of severe somatic illness or death in the family from 0–5 years, accompanied by slightly elevated rates of material deprivation. Less than two percent (1.7%) of children was in the *high adversity* group characterized by high rates of adversity in the family dynamics dimension, while being exposed to the same rate of material deprivation as children in the moderate material deprivation group.

Other characteristics of these five adversity groups are shown in Supplementary Table 3. There were relatively more children with a parent with a non-Western background in the moderate material deprivation group (15%), high material deprivation group (28%) and the loss or threat of loss group (21%) than in the low and high adversity group (both 6%). In addition, there were relatively more children with low parental education in the high adversity (46%), high material deprivation (35%), moderate material deprivation (27%) and the loss or threat of loss group (24%) than in the low adversity group (14%). The differences in mean BMI z-scores between the different childhood adversity groups were rather small (Supplementary Table 4).

### Childhood adversity and BMI

**Fig. 3:**
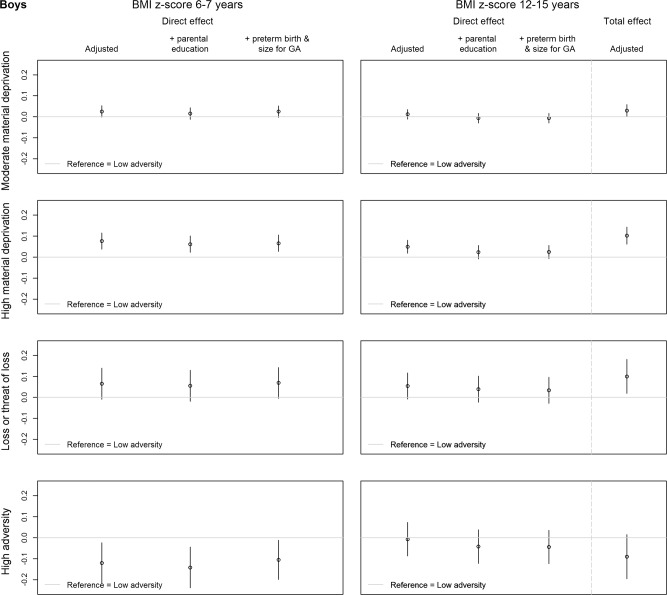
Effect estimates and 95% CI of the direct and total association between the early childhood adversity groups and BMI z-score at 6–7 years (childhood) and 12–15 years (adolescence), compared with the low adversity group, for boys (*n* = 27,241). The direct and total association of the adjusted model (adjusted for parental origin, maternal age at birth, parental cardiometabolic illness, and birth year) are shown. The direct association with BMI z-score in adolescence is also adjusted for BMI z-score in childhood. Additionally, the direct associations in the models additionally adjusted for parental education at birth, and additionally adjusted for size for gestational age and preterm birth are displayed.

**Fig. 4:**
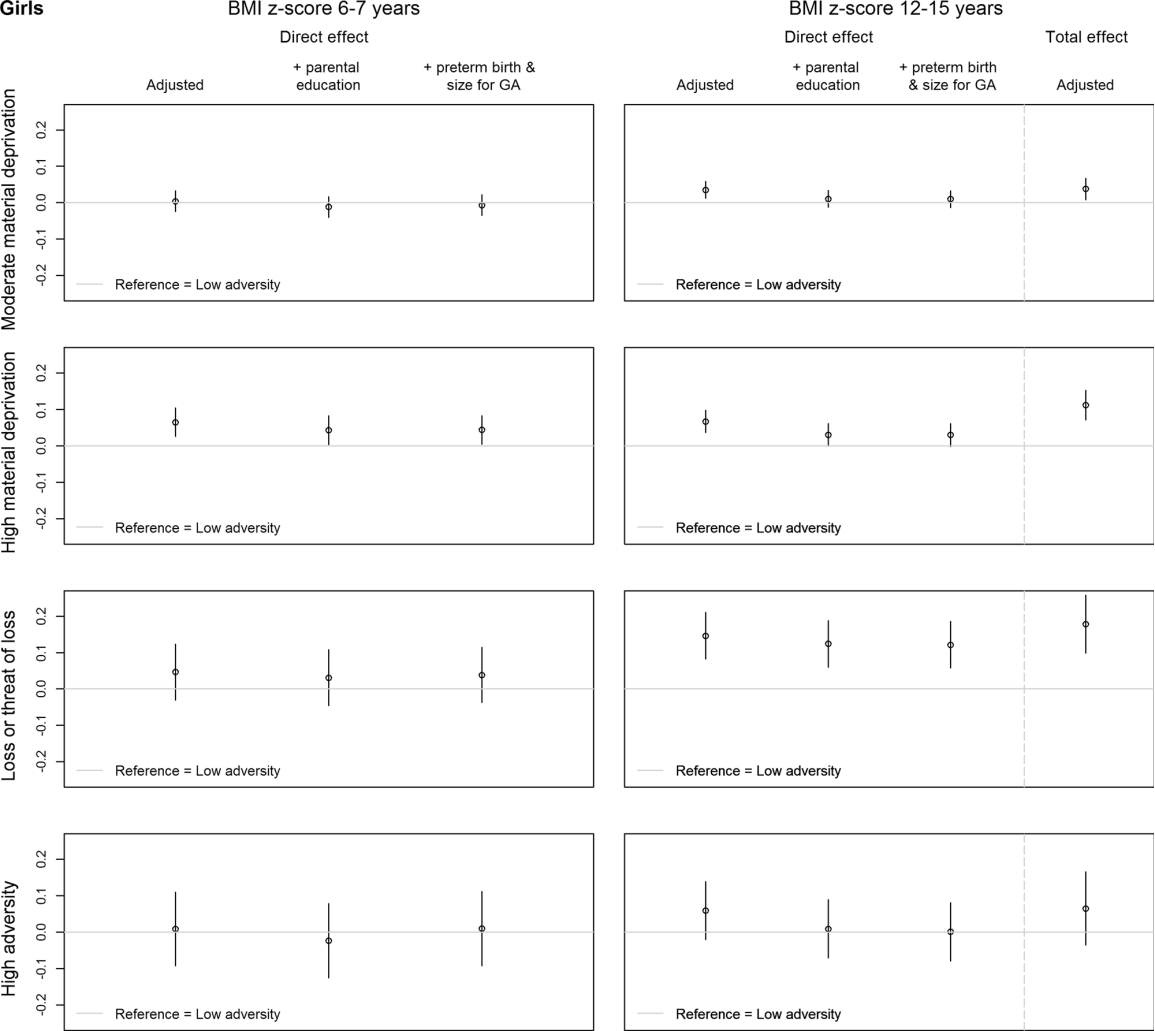
Effect estimates and 95% CI of the direct and total association between the early childhood adversity groups and BMI z-score at 6–7 years (childhood) and 12–15 years (adolescence), compared with the low adversity group, for girls (*n* = 26,160). The direct and total association of the adjusted model (adjusted for parental origin, maternal age at birth, parental cardiometabolic illness, and birth year) are shown. The direct association with BMI z-score in adolescence is also adjusted for BMI z-score in childhood. Additionally, the direct associations in the models additionally adjusted for parental education at birth, and additionally adjusted for size for gestational age and preterm birth are displayed.

Figures 3 and 4 show the direct and total association of the early childhood adversity groups with BMI in childhood (6-7 years) and adolescence (12–15 years) for boys and girls, respectively, using the low adversity group as a reference. The direct associations with adolescent BMI are adjusted for childhood BMI (Fig. 1b). For every association, the coefficients of the main analysis (adjusted for parental origin, maternal age at birth, parental cardiometabolic illness, and birth year), and the additional analyses (further adjusted for parental education at birth, and for size for gestational age and preterm birth) are shown. The associations are virtually identical in the three different models. The estimates of the main analysis are reported below. Full results of the analysis adjusted for all possible confounders can be found in Supplementary Table 5 (boys) and 6 (girls).

Boys in the *high material deprivation group* had a slightly higher BMI z-score in childhood compared with boys in the *low adversity group* (b (95% CI) = 0.08 (0.04, 0.12)). In contrast, boys in the *high adversity group* had a slightly lower BMI z-score in childhood than boys in the *low adversity group* (b (95% CI) = −0.12 (−0.22, −0.02)). There was a tendency for boys in the *moderate material deprivation group* (b (95% CI) = 0.03 (0.00, 0.05)) and *loss or threat of loss group* (b (95% CI) = 0.07 (−0.01, 0.14)) to have a slightly higher BMI z-score in childhood than boys in the *low adversity group*, but the confidence intervals overlap with zero (Fig. 3).

The direct associations between the childhood adversity groups and BMI z-score in adolescence among boys resembled the associations in childhood for all groups, except for the high adversity group, but the estimates were generally lower. The estimates for the total effect in adolescence, comprising both the direct effect and the indirect effect through childhood BMI, showed that the *moderate material deprivation group* (b (95% CI) = 0.03 (0.00, 0.06)), *high material deprivation group* (b (95% CI) = 0.10 (0.06, 0.14)), and *loss or threat of loss group* (b (95% CI) = 0.10 (0.02, 0.18)), compared with the *low adversity group*, were associated with a slightly higher BMI z-score in adolescence (Fig. 3). To exemplify the clinical relevance of these differences, the difference in weight between a BMI z-score of ‘0’ and ‘0.10’, when comparing two boys of 14 years with an average height, would be 0.8 kg (1.5% of the weight of the first).

Girls in the *high material deprivation group* had a slightly higher BMI z-score in childhood compared with girls in the *low adversity group* (b (95% CI) = 0.07 (0.03, 0.10)). This difference seemed to get more pronounced in adolescence, as shown by the estimate of the direct association with BMI in adolescence (b (95% CI) = 0.07 (0.04, 0.10)). The BMI of girls in the *moderate material deprivation group* (b (95% CI) = 0.04 (0.01, 0.06)) and *loss or threat of loss group* (b (95% CI) = 0.15 (0.08, 0.21)) became higher than the BMI of girls in the *low adversity group* between childhood and adolescence. No other direct associations between the childhood adversity groups and BMI in childhood or adolescence were identified among girls. The total association estimates indicated an association between the *moderate material deprivation group* (b (95% CI) = 0.04 (0.01, 0.07)), the *high material deprivation* (b (95% CI) = 0.11 (0.07, 0.15)) and the *loss or threat of loss group* (b (95% CI) = 0.18 (0.10, 0.26)), compared with the *low adversity group*, with BMI in adolescence (Fig. 4). Again to exemplify the clinical relevance, when comparing two girls of 14 years with an average height and a BMI z-score of ‘0’ and ‘0.18’, the difference in weight would be 1.5 kg (2.8% of the weight of the first).

The effect estimates in the sensitivity analysis, which was adjusted for parental origin, maternal age at birth and birth year, but not for parental cardiometabolic illness in the three years before birth, were virtually identical to the effect estimates in the main analysis (Supplementary Tables 7 and 8).

## Discussion

In this study, we examined prospective associations between trajectories of adversity in early childhood and BMI at school entry and in adolescence using a large, unselected sample from Copenhagen. We identified five adversity groups in early childhood, which were characterized by low adversity, moderate material deprivation, high material deprivation, loss or threat of loss, and high adversity. Overall, the results did not support a strong relationship between early life adversity and childhood and adolescent BMI. However, the patterns of associations differed across childhood adversity groups. We found that both boys and girls who had been exposed to moderate or high material deprivation or who had been exposed to loss or threat of loss were more likely to have a higher BMI in adolescence than children in the low adversity group. This association was already present at school entry age for children exposed to high material deprivation. A small group of children were exposed to high levels of adversity across different dimensions, and contrary to our hypothesis, we found no clear associations with BMI in this group.

In line with a previous study assessing the association between adverse childhood experiences and obesity in 17-year olds in England [25], we did not identify strong associations between childhood adversity and BMI. Given that the risk estimates are quite small, they may only be of limited clinical relevance. However, because the differences seem to increase across age, and BMI tends to track from adolescence into adulthood, these small differences may eventually translate into relevant differences in BMI and associated morbidity later in life [6, 26]. In addition, adversity is also likely to track from early childhood into later life [27]. This means that children exposed to early life adversity are more likely to be exposed to additional adversity in adolescence, and in adulthood. This notion is supported by the adversity groups we have previously identified based on data from the entire childhood period (0–15 years) [2].

The findings suggest that the associations may differ across dimensions of adversity. While exposure to material deprivation and to loss or threat of loss were related to a slightly higher BMI in the current study, exposure to adversity in the family dynamics dimension seemed related to a slightly lower BMI in boys. Different types of childhood adversity are suggested to have different physiological and health consequences [28], and both overweight and underweight may be problematic for health and well-being in later life. The finding that different types of adversities, and different combinations of adversity, are differently associated with health [29] and BMI [10, 11, 18, 30] is in line with previous studies. This finding may be explained by the specific effects of childhood adversity, as well as by the severity of the childhood adversity experienced. Material deprivation may result in less resources in the family, and less opportunities for healthy eating or physical activity. For example, poverty has been shown to be associated with lower-quality diets [31]. Loss or threat of loss may specifically have an effect on mental health, available resources, material as well as social support, and may be particularly stressful [32]. High adversity resulting from a dysfunctional family has previously been hypothesized to be related to lower BMI due to a combination of chronic stress and low resources, which could explain the finding in childhood among boys in this group [33]. However, persistent poverty and poor parental mental health have also been related to a higher risk of obesity in 14-year-old children [18].

Overall, similar associations were identified among boys and girls. A previous systematic review examined the association between adverse events and obesity in childhood. It included a mix of studies on childhood maltreatment and a broader range of adverse childhood experiences and the majority were performed in the USA. The conclusion of the review was that girls may be more sensitive than boys to effects of adverse events related to obesity [9]. We did find that the association between the loss or threat of loss group, compared with the low adversity group, and BMI in adolescence was specifically present and pronounced among girls, and that the association between the high adversity group, compared with the low adversity group, was specific for boys. These differences could be indicative of different effects of childhood adversity on BMI of boys and girls, but do not provide clear evidence of sex differences.

### Strengths and limitations

Major strengths of this study include the combined use of nationwide register data and a population-based cohort. The applied registers contained objective, yearly data on 12 different childhood adversities, and on height and weight of children born in Denmark who attended public and private schools in Copenhagen. As such, recall bias is not an issue in the current study, and temporal information on childhood adversities is available. This temporal information was used to define the adversity groups in line with the *accumulation of risk* hypothesis [34], while taking into account the *timing* of adversity and the *duration* of adversity. As schooling outside of public and private schools is rare in Denmark, information on height and weight on nearly all school children in the Copenhagen area was available [16]. The height and weight data available also allowed for the creation of age- and sex-specific reference curves for BMI. As such, the associations identified in this study are not influenced by the suitability of external reference curves for our population at different ages. Finally, the data allowed examination of the associations between early childhood adversity and BMI in both childhood and adolescence, which enabled us to generate a complete picture of these longitudinal associations.

While the comprehensive adversity groups are a particular strength of this study, we also acknowledge that dividing individuals into groups carries limitations. The children in the different groups follow approximately the same trajectory of adversity over childhood [17], but there is uncertainty around the groups as well as around children’s assignment to the groups. This could influence the identification and size of effects, particularly among the smaller groups. In post-hoc analyses weighted by children’s posterior probabilities, we found similar results as in our main analysis, though for boys some of the associations were somewhat weaker (Supplementary Tables 9 and 10).

Although a wide range of childhood adversities in different dimensions are included in the current study, no direct measures of violence in the household or childhood maltreatment were available. As we included childhood adversities that are related to family violence and maltreatment (e.g., being placed in foster care), we believe that the identified groups capture important differences in the exposure to childhood adversity. Previously, we have shown that a similar categorization of childhood adversity exposure from 0–15 years was related to mortality in early adulthood [2]. Further, confounding, by for example genetics or neighbourhood characteristics, cannot be ruled out in this study. Finally, it is important to note that because we examined the association between childhood adversity with BMI at 6–7 years, and at 12–15 years, the average time between the BMI measurements in childhood and adolescence will be around 7 years, but for some children it will only be 4 years. This may have influenced our ability to identify direct associations with adolescent BMI.

## Conclusion

The findings from this large population-based study show small associations between early childhood adversity and BMI in adolescence. Associations depended upon the type and severity of the childhood adversities experienced, which indicates that different types of childhood adversity exposure may differentially impact weight and health. Early preventative efforts may be warranted to prevent childhood adversity and adverse health in childhood and later in life, but BMI in childhood and adolescence generally does not seem to be an important factor linking early adversity with later-life health.

### Supplementary information

Supplementary Information

## Data Availability

The data material contains personally identifiable and sensitive information, and can therefore not be made publicly available. Inquiries about secure access to the DANLIFE data under conditions stipulated by the Danish Data Protection Agency can be directed at principal investigator of the study Naja Hulvej Rod (nahuro@sund.ku.dk) and inquiries about access to the CSHRR can be directed at the CSHRR steering committee (CSHRR@regionh.dk) at The Center for Clinical Research and Prevention, Copenhagen University Hospital - Bispebjerg and Frederiksberg, Capital Region, Denmark that governs the use of these data.
