# Supplementary material for: Early childhood adversity and body mass index in childhood and adolescence: linking registry data on adversities with school health records of 53,401 children from Copenhagen
Source: Int J Obes (Lond). 2023 Aug 25;47(11):1057–64. doi: 10.1038/s41366-023-01355-9 (PMC10599995; doi:10.1038/s41366-023-01355-9)
Supplement: Supplementary file 1 — Supplementary Information [file 41366_2023_1355_MOESM1_ESM.pdf]

Supplementary Table S1. Definitions of the twelve included childhood adversities listed per dimension with the used register.

All childhood adversities were allowed to occur once for each parent or sibling per year of life of the child. A count was determined per dimension and year of life of the child.

Material deprivation	
1. *Family poverty* - Family income below 50% of the median national family income in a given year	The Income Statistics Register[a]
2. *Parental long-term unemployment* - Unemployment of a parent for at least 12 months in the period of two consecutive years	The Integrated Database for Labour Market Research
Loss or threat of loss	
3. *Death of a parent* - Death of a parent	The Danish Civil Registration System
4. *Death of a sibling* - Death of a sibling	The Danish Civil Registration System
5. *Parental somatic illness* - Diagnosis of a parent with one of the diseases included in the Charlson comorbidity index	The Danish National Patient Registry
6. *Sibling somatic illness* - Diagnosis of a sibling with one of the seven most common somatic diseases related to mortality in children of 0-18 years of age in Denmark	The Danish National Patient Registry
Family dynamics	
7. *Foster care placement* - Being in out-of-home care	The Register of Support for Children and Adolescents
8. *Parental psychiatric illness* - Hospitalization of a parent with a main diagnosis related to psychiatric illness, excluding main diagnoses related to alcohol and drug abuse	The Danish Psychiatric Central Research Register; The Danish National Patient Register
9. *Sibling psychiatric illness* - Hospitalization of a sibling with a main diagnosis related to psychiatric illness	The Danish Psychiatric Central Research Register; The Danish National Patient Register
10. *Parental alcohol abuse* - Diagnosis of a parent with a disease related to alcohol abuse or a parent getting a prescribed drug that is used in treatment of alcohol addiction	The Danish Psychiatric Central Research Register; The Danish National Patient Register; The Danish National Prescription Registry
11. *Parental drug abuse* - Diagnosis of a parent with a disease related to drug abuse or a parent getting a prescribed drug that is used in treatment of drug addiction	The Danish Psychiatric Central Research Register; The Danish National Patient Register; The Danish National Prescription Registry
12. *Maternal separation* - The mother no longer sharing an address with a partner	The Danish Civil Registration System

[a]Data available from 1987 onwards.

Supplementary Table S2. Model adequacy and fit statistics of the 4, 5 and 6 group model.

**Number of groups**	4	5	6
**Average posterior probability**			
Low adversity	0.89	0.89	0.48
Moderate material deprivation	0.76	0.77	0.75
High material deprivation	0.81	0.81	0.80
High adversity	0.85	0.88	0.89
Loss or threat of loss		0.77	0.78
Occasional adversity			0.75
**BIC – based on no. data points**[a]	-301551.97	-300217.45	-300257.93
**BIC – based on sample size**[a]	-301443.58	-300081.60	-300094.62
**Group membership probability (SE)**			
Low adversity	47.2 (0.37)	46.4 (0.34)	17.8 (1.06)
Moderate material deprivation	31.4 (0.52)	30.5 (0.51)	30.2 (0.49)
High material deprivation	17.6 (0.59)	17.2 (0.55)	16.5 (0.56)
High adversity	3.8 (0.16)	2.2 (0.10)	2.0 (0.09)
Loss or threat of loss		3.7 (0.20)	3.7 (0.18)
Occasional adversity			29.8 (1.09)

[a] The best fitting model according to the BIC is the one where the BIC is closest to zero.[1–3]

Supplementary Table S3. Background characteristics of the study population (n=53,401) according to the five childhood adversity groups.

	Low adversity	Moderate material deprivation	High material deprivation	Loss or threat of loss	High adversity
	**n = 27,413**	**n = 16,046**	**n = 7,436**	**n = 1,597**	**n = 909**
**Sex**					
Boys	51%	51%	51%	50%	51%
Girls	49%	49%	49%	50%	49%
Missing	0%	0%	0%	0%	0%
**Parental origin**					
Non-Western	6%	15%	28%	21%	6%
Western	94%	85%	72%	79%	94%
Missing	<1%	<1%	<1%	<1%	0%
**Maternal age (years)**					
<20	3%	6%	7%	4%	8%
20 - 30	63%	70%	69%	61%	62%
>30	34%	24%	24%	35%	30%
Missing	<1%	<1%	<1%	0%	0%
**Parental cardiometabolic disease[a]**					
No	99%	99%	99%	92%	98%
Yes	1%	1%	1%	8%	2%
Missing	0%	0%	0%	0%	0%
**Parental education**					
Low	14%	27%	35%	24%	46%
Medium	40%	43%	40%	43%	36%
High	45%	28%	23%	32%	18%
Missing	<1%	1%	2%	1%	<1%
**Size for gestational age**					
Small	14%	16%	16%	17%	24%
Average	74%	74%	74%	70%	66%
Large	9%	8%	8%	11%	7%
Missing	2%	2%	2%	2%	3%
**Preterm**					
No	93%	93%	92%	91%	89%
Yes	5%	5%	6%	7%	8%
Missing	2%	2%	2%	2%	3%

[a]In the three years before birth of the child.

Supplementary Table S4. BMI z-scores of boys and girls in the different childhood adversity groups in childhood and adolescence with the percentage of missing data (n=53,401). The BMI z-scores are displayed as mean (SD).

	Low adversity	Moderate material deprivation	High material deprivation	Loss or threat of loss	High adversity	Overall
Boys	**n = 14,049**	**n = 8,151**	**n = 3,778**	**n = 801**	**n = 462**	**n = 27,241**
BMI z-score childhood	-0.04 (0.95)	0.00 (0.99)	0.05 (1.07)	0.04 (1.01)	-0.14 (1.02)	-0.01 (0.98)
Missing	6.5%	7.3%	8.1%	7.2%	7.4%	7.0%
BMI z-score adolescence	-0.04 (0.94)	0.00 (1.00)	0.10 (1.06)	0.06 (1.04)	-0.14 (1.04)	-0.01 (0.98)
Missing	22.5%	24.9%	22.8%	27.5%	30.7%	23.5%
Girls	**n = 13,364**	**n = 7,895**	**n = 3,658**	**n = 796**	**n = 447**	**n = 26,160**
BMI z-score childhood	-0.03 (0.95)	-0.02 (1.00)	0.04 (1.04)	0.03 (1.05)	-0.01 (1.06)	-0.02 (0.99)
Missing	6.9%	7.3%	8.7%	9.4%	9.4%	7.4%
BMI z-score adolescence	-0.04 (0.94)	0.01 (0.99)	0.12 (1.04)	0.16 (1.04)	0.05 (0.98)	0.01 (0.98)
Missing	21.4%	24.0%	21.5%	25.9%	30.6%	22.5%

BMI z-scores are based on reference curves generated using data from the Copenhagen School Health Records Register and the LMS method.
At every age the BMI z-scores of boys and girls should have a mean of '0' and a standard deviation of '1'.

Supplementary Table S5. Effect estimates and 95% CI of the direct association between the early childhood adversity groups and BMI z-score at 6-7 years (childhood) and 12-15 years (adolescence), compared with the low adversity group, for boys. The model is adjusted for parental origin, maternal age at birth, parental cardiometabolic illness, birth year, parental education at birth, size for gestational age and preterm birth.

	**b**	**95% CI**	
		**Lower bound**	**Upper bound**
**BMI z-score childhood**			
Early childhood adversity groups (ref: Low adversity)			
Moderate material deprivation	0.025	-0.003	0.052
High material deprivation	0.066	0.027	0.105
Loss or threat of loss	0.069	-0.004	0.142
High adversity	-0.105	-0.199	-0.011
Parental origin (ref: non-Western background)			
Western background	0.019	-0.023	0.061
Maternal age (ref: average)			
Younger	0.083	0.025	0.142
Older	-0.071	-0.098	-0.043
Parental cardiometabolic illness (ref: no)			
Yes	0.164	0.027	0.300
Birth year	0.007	0.004	0.009
Parental education (ref: medium)			
Low	0.031	-0.002	0.065
High	-0.073	-0.100	-0.045
Size for gestational age (ref: average)			
Small	-0.328	-0.364	-0.291
Large	0.329	0.290	0.368
Preterm birth (ref: no)			
Yes	-0.120	-0.174	-0.065
**BMI z-score adolescence**			
BMI z-score childhood	0.692	0.680	0.704
Early childhood adversity groups (ref: Low adversity)			
Moderate material deprivation	-0.006	-0.030	0.017
High material deprivation	0.025	-0.007	0.056
Loss or threat of loss	0.034	-0.028	0.096
High adversity	-0.044	-0.124	0.036
Parental origin (ref: non-Western background)			
Western background	-0.062	-0.097	-0.026
Maternal age (ref: average)			
Younger	0.034	-0.017	0.084
Older	-0.014	-0.037	0.008
Parental cardiometabolic illness (ref: no)			
Yes	0.009	-0.102	0.120
Birth year	0.001	-0.001	0.003
Parental education (ref: medium)			
Low	0.044	0.016	0.073
High	-0.092	-0.115	-0.069
Size for gestational age (ref: average)			
Small	0.044	0.014	0.074
Large	0.029	-0.005	0.063
Preterm birth (ref: no)			
Yes	0.037	-0.006	0.080

Supplementary Table S6. Effect estimates and 95% CI of the direct association between the early childhood adversity groups and BMI z-score at 6-7 years (childhood) and 12-15 years (adolescence), compared with the low adversity group, for girls. The model is adjusted for parental origin, maternal age at birth, parental cardiometabolic illness, birth year, parental education at birth, size for gestational age and preterm birth.

	**b**	**95% CI**	
		**Lower bound**	**Upper bound**
**BMI z-score childhood**			
Early childhood adversity groups (ref: Low adversity)			
Moderate material deprivation	-0.007	-0.035	0.022
High material deprivation	0.044	0.004	0.083
Loss or threat of loss	0.039	-0.038	0.115
High adversity	0.009	-0.093	0.111
Parental origin (ref: non-Western background)			
Western background	0.035	-0.007	0.077
Maternal age (ref: average)			
Younger	0.043	-0.015	0.100
Older	-0.022	-0.050	0.005
Parental cardiometabolic illness (ref: no)			
Yes	0.025	-0.105	0.156
Birth year	0.007	0.004	0.009
Parental education (ref: medium)			
Low	0.041	0.007	0.074
High	-0.098	-0.126	-0.069
Size for gestational age (ref: average)			
Small	-0.297	-0.332	-0.263
Large	0.337	0.293	0.381
Preterm birth (ref: no)			
Yes	-0.085	-0.144	-0.026
**BMI z-score adolescence**			
BMI z-score childhood	0.693	0.682	0.705
Early childhood adversity groups (ref: Low adversity)			
Moderate material deprivation	0.010	-0.013	0.033
High material deprivation	0.030	-0.002	0.061
Loss or threat of loss	0.121	0.058	0.185
High adversity	0.001	-0.080	0.081
Parental origin (ref: non-Western background)			
Western background	-0.147	-0.182	-0.113
Maternal age (ref: average)			
Younger	0.068	0.017	0.120
Older	-0.015	-0.037	0.007
Parental cardiometabolic illness (ref: no)			
Yes	0.085	-0.026	0.195
Birth year	-0.006	-0.008	-0.004
Parental education (ref: medium)			
Low	0.092	0.065	0.120
High	-0.095	-0.117	-0.072
Size for gestational age (ref: average)			
Small	0.060	0.032	0.088
Large	-0.005	-0.042	0.032
Preterm birth (ref: no)			
Yes	0.052	0.004	0.099

Supplementary Table S7. Effect estimates and 95% CI of the direct and total associations between the early childhood adversity groups and BMI z-score at 6-7 years (childhood) and 12-15 years (adolescence), compared with the low adversity group, for boys. The model is adjusted for parental origin, maternal age at birth, and birth year.

				95% CI	
	**Coef.**	**St. Error**	**p**	**Lower**	**Upper**
**BMI z-score childhood**					
Moderate material deprivation	0.025	0.014	0.072	-0.002	0.053
High material deprivation	0.077	0.020	0.000	0.038	0.116
Loss or threat of loss	0.076	0.038	0.043	0.002	0.151
High adversity	-0.118	0.049	0.017	-0.214	-0.021
**BMI z-score adolescence**					
Moderate material deprivation	0.012	0.012	0.316	-0.011	0.035
High material deprivation	0.050	0.016	0.002	0.018	0.081
Loss or threat of loss	0.056	0.032	0.076	-0.006	0.119
High adversity	-0.007	0.041	0.872	-0.086	0.073
**Total**					
Moderate material deprivation	0.029	0.015	0.050	0.000	0.059
High material deprivation	0.103	0.021	0.000	0.062	0.144
Loss or threat of loss	0.109	0.041	0.008	0.029	0.190
High adversity	-0.088	0.054	0.103	-0.194	0.018

Ref = low adversity

Supplementary Table S8. Effect estimates and 95% CI of the direct and total associations between the early childhood adversity groups and BMI z-score at 6-7 years (childhood) and 12-15 years (adolescence), compared with the low adversity group, for girls. The model is adjusted for parental origin, maternal age at birth, and birth year.

				95% CI	
	**Coef.**	**St. Error**	**p**	**Lower**	**Upper**
**BMI z-score childhood**					
Moderate material deprivation	0.004	0.014	0.796	-0.025	0.032
High material deprivation	0.065	0.020	0.001	0.026	0.105
Loss or threat of loss	0.051	0.039	0.189	-0.025	0.128
High adversity	0.009	0.052	0.867	-0.093	0.110
**BMI z-score adolescence**					
Moderate material deprivation	0.035	0.012	0.003	0.012	0.058
High material deprivation	0.068	0.016	0.000	0.037	0.099
Loss or threat of loss	0.152	0.033	0.000	0.088	0.216
High adversity	0.059	0.041	0.148	-0.021	0.138
**Total**					
Moderate material deprivation	0.037	0.015	0.013	0.008	0.067
High material deprivation	0.113	0.021	0.000	0.072	0.154
Loss or threat of loss	0.188	0.041	0.000	0.108	0.267
High adversity	0.065	0.051	0.207	-0.036	0.166

Ref = low adversity

Supplementary Table S9. Effect estimates and 95% CI of the direct and total associations between the early childhood adversity groups and BMI z-score at 6-7 years (childhood) and 12-15 years (adolescence), compared with the low adversity group, for boys. The model is adjusted for parental origin, maternal age at birth, parental cardiometabolic illness, and birth year and weighted by children's posterior probabilities.

				95% CI	
	**Coef.**	**St. Error**	**p**	**Lower**	**Upper**
**BMI z-score childhood**					
Moderate material deprivation	0.025	0.011	0.023	0.003	0.047
High material deprivation	0.062	0.015	0.000	0.032	0.092
Loss or threat of loss	0.044	0.025	0.086	-0.006	0.093
High adversity	-0.070	0.036	0.056	-0.141	0.002
**BMI z-score adolescence**					
Moderate material deprivation	0.011	0.009	0.234	-0.007	0.029
High material deprivation	0.036	0.012	0.003	0.012	0.060
Loss or threat of loss	0.040	0.021	0.065	-0.002	0.082
High adversity	0.001	0.031	0.970	-0.059	0.061
**Total**					
Moderate material deprivation	0.029	0.012	0.016	0.005	0.052
High material deprivation	0.079	0.016	0.000	0.048	0.110
Loss or threat of loss	0.070	0.028	0.012	0.016	0.124
High adversity	-0.047	0.040	0.237	-0.125	0.031

Ref = low adversity

Supplementary Table S10. Effect estimates and 95% CI of the direct and total associations between the early childhood adversity groups and BMI z-score at 6-7 years (childhood) and 12-15 years (adolescence), compared with the low adversity group, for girls. The model is adjusted for parental origin, maternal age at birth, parental cardiometabolic illness, and birth year and weighted by children's posterior probabilities.

				**95% CI**	
	**Coef.**	**St. Error**	**p**	**Lower**	**Upper**
**BMI z-score childhood**					
Moderate material deprivation	0.010	0.012	0.401	-0.013	0.032
High material deprivation	0.055	0.015	0.000	0.025	0.086
Loss or threat of loss	0.044	0.027	0.099	-0.008	0.097
High adversity	0.008	0.038	0.838	-0.066	0.082
**BMI z-score adolescence**					
Moderate material deprivation	0.031	0.009	0.001	0.013	0.049
High material deprivation	0.063	0.012	0.000	0.039	0.087
Loss or threat of loss	0.127	0.022	0.000	0.085	0.170
High adversity	0.068	0.029	0.021	0.010	0.125
**Total**					
Moderate material deprivation	0.038	0.012	0.002	0.014	0.061
High material deprivation	0.102	0.016	0.000	0.070	0.133
Loss or threat of loss	0.158	0.027	0.000	0.105	0.211
High adversity	0.073	0.037	0.047	0.001	0.145

Ref = low adversity

References

1. Klijn SL, Weijenberg MP, Lemmens P, Van Den Brandt PA, Lima Passos V. Introducing the fit-criteria assessment plot-A visualisation tool to assist class enumeration in group-based trajectory modelling. *Stat Methods Med Res*. 2017;26(5):2424-2436.

2. Nagin DS, Jones BL, Passos VL, Tremblay RE. Group-based multi-trajectory modeling. *Stat Methods Med Res*. 2018;27(7):2015-2023.

3. Adjei NK, Schlüter DK, Straatmann VS, et al. Impact of poverty and family adversity on adolescent health: a multi-trajectory analysis using the UK Millennium Cohort Study. *Lancet Reg Heal - Eur*. 2022;13:100279.
